# Role of Catecholate Siderophores in Gram-Negative Bacterial Colonization of the Mouse Gut

**DOI:** 10.1371/journal.pone.0050020

**Published:** 2012-11-29

**Authors:** Hualiang Pi, Shari A. Jones, Lynn E. Mercer, Jessica P. Meador, Joyce E. Caughron, Lorne Jordan, Salete M. Newton, Tyrrell Conway, Phillip E. Klebba

**Affiliations:** 1 Department of Chemistry and Biochemistry, University of Oklahoma, Stevenson Life Sciences Research Center, Norman, Oklahoma, United States of America; 2 Department of Microbiology and Plant Biology, University of Oklahoma, Stevenson Life Sciences Research Center, Norman, Oklahoma, United States of America; 3 Department of Biochemistry, Kansas State University, Manhattan, Kansas, United States of America; Charité, Campus Benjamin Franklin, Germany

## Abstract

We investigated the importance of the production of catecholate siderophores, and the utilization of their iron (III) complexes, to colonization of the mouse intestinal tract by *Escherichia coli*. First, a *ΔtonB* strain was completely unable to colonize mice. Next, we compared wild type *E. coli* MG1655 to its derivatives carrying site-directed mutations of genes for enterobactin synthesis (*ΔentA::Cm;* strain CAT0), ferric catecholate transport (*Δfiu, ΔfepA, Δcir, ΔfecA::Cm*; CAT4), or both (*Δfiu, ΔfepA, ΔfecA, Δcir, ΔentA::Cm;* CAT40) during colonization of the mouse gut. Competitions between wild type and mutant strains over a 2-week period *in vivo* showed impairment of all the genetically engineered bacteria relative to MG1655. CAT0, CAT4 and CAT40 colonized mice 10^1^-, 10^5^-, and 10^2^-fold less efficiently, respectively, than MG1655. Unexpectedly, the additional inability of CAT40 to synthesize enterobactin resulted in a 1000-fold better colonization efficiency relative to CAT4. Analyses of gut mucus showed that CAT4 hyperexcreted enterobactin *in vivo*, effectively rendering the catecholate transport-deficient strain iron-starved. The results demonstrate that, contrary to prior reports, iron acquisition via catecholate siderophores plays a fundamental role in bacterial colonization of the murine intestinal tract.

## Introduction

For most microorganisms and all animals, iron is indispensable in metabolic processes like catabolism, electron transport, peroxide reduction, and DNA biosynthesis. More than 100 structurally-solved enzymes contain the metal in heme groups or iron-sulfur clusters [1]. The fact that iron readily oxidizes in aqueous environments poses a problem for its acquisition by living organisms: cells cannot transport the large polymers of insoluble ferric oxyhydroxide that spontaneously form in water. Therefore, almost all microorganisms, including commensal and pathogenic bacteria, produce biosynthetic and transport systems to capture the metal from their environments, including their plant and animal hosts. Mammalian body fluids and tissues have low iron availability, in part from its poor solubility at physiological pH (the concentration of free iron in neutral aqueous solutions is ∼10^−18^ M [2]), and in part because binding proteins like transferrin, lactoferrin, ferritin and hemoproteins complex Fe^+++^, reducing the level of free iron to ∼10^−24^ M [3], [4]. Furthermore, mammals compensate increases in dietary iron by reducing intestinal iron absorption or assimilation [5] and simultaneously increasing levels of serum transferrin and cellular ferritin. The sequestration of iron from invading pathogens was described as “nutritional immunity,” even though no immune system components are involved in the process [6].

Microorganisms circumvent low iron availability by at least four methods. First, they may directly utilize available extracellular ferrous iron [7], [8]. Secondly, they synthesize and secrete siderophores [9] that complex ferric iron with high affinity and may thereby remove it from mammalian iron-binding proteins. Next, prokaryotic cells may directly bind host iron-containing molecules (ferritin, transferrin, lactoferrin and hemoglobin) on their surfaces, where membrane receptor proteins extract the metal [10], [11], [12]. Lastly, pathogenic microbes may directly transport free heme [12], [13], [14] or use hemophores to remove the porphyrin from hemoglobin, hemoglobin-haptoglobin or hemopexin [15]. So, from the perspective of the opportunistic bacteria the concentration of available iron is much higher than 10^−24^–10^−18^ M, as a result of their diverse mechanisms to overcome mammalian iron sequestration. For example, the total concentration of iron in human blood is 10 mM (in a 5 L volume, 2.5 g of hemoglobin), creating an iron-replete environment for pathogens with mechanisms to obtain it.

As a result of iron's central biochemical role, its acquisition in the host environment is a determinant of bacterial virulence (for review see [16]), and inhibition of iron uptake combats bacterial infections. Strains that cannot obtain iron in mammalian hosts may therefore have potential as attenuated live vaccines [17]. Nevertheless, these concepts remain controversial as a result of conflicting data, and little is known about what forms of iron are available and utilized by enteric bacteria during colonization of the host gut. *E. coli* K-12 strain MG1655 colonizes the mouse intestine despite the presence of other microbiota, reaching a population of about 10^8^ colony forming units (CFU)/g of feces. The native siderophores of Gram-negative bacteria are often catecholate compounds like enterobactin and its glycosylated form (also called salmochelin) [9]. To investigate the impact of catecholate siderophore-mediated iron uptake on bacterial colonization of mice we constructed chromosomal deletions in *E. coli* MG1655 and performed competition experiments with the resulting strains. Besides the wild-type, we genetically engineered four test strains that discriminated between siderophore production and utilization during colonization. One (*E. coli* CAT0; *ΔentA::Cm*) did not synthesize enterobactin, the native *E.coli* siderophore. Another (*E. coli* CAT4; *Δfiu*, *ΔfepA, Δcir, ΔfecA::Cm*,) lacked all outer membrane (OM) receptors for ferric catecholate transport. A third (*E. coli* CAT40) combined all the deletions, making it deficient in both enterobactin biosynthesis and ferric catecholate transport. Ferric catecholate uptake in Gram-negative bacteria requires the function of the additional cell envelope protein TonB [18], and we also studied colonization by a *ΔtonB::Cm* strain (*E. coli Δ*TonB) in the mouse model system. The results show the need for bacterial iron acquisition in the mouse gut to achieve colonization; toward that end *E. coli* secretes enterobactin and transports ferric enterobactin (FeEnt) in the host environment.

## Results

### Construction of multiple deletion strains

**Figure 1:**
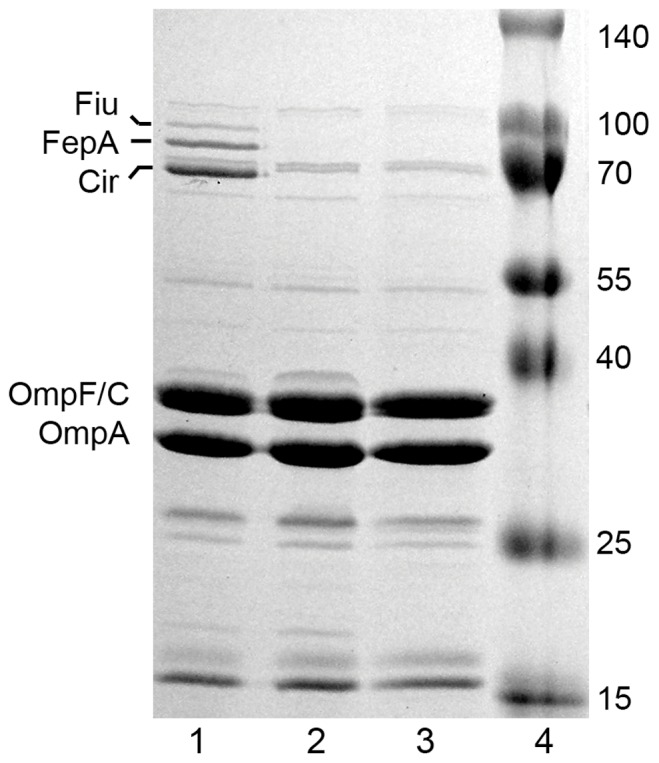
SDS-PAGE of OM fractions. The prototypic *E. coli* strain MG1655 and its derivatives CAT4 (*Δfiu, ΔfepA, Δcir, ΔfecA::Cm*) and CAT40 (*Δfiu, ΔfepA, Δcir, ΔfecA, ΔentA::Cm*) were grown in LB broth, subcultured at 1% into iron-deficient MOPS minimal media at 37°C and grown to late log phase. The bacteria were collected by centrifugation, lysed in a French pressure cell and their OM fractions were purified, resolved by SDS-PAGE, and the gels were stained with coomassie blue R. Fiu, FepA and Cir are seen in MG1655 (Lane 1), but absent from CAT4 (lane 2) and CAT40 (lane 3). Molecular weight standards were included in lane 4. FecA, which is inducible by growth in the presence of citrate, is not visible in this experiment, but its absence was verified by PCR.

We previously constructed [19] five strains that contained site-directed deletions in iron transport loci (Table 1; [20]) in *E. coli* strain BN1071. We moved these markers to *E. coli* MG1655 by P1 transduction. *E. coli* CAT0 (*ΔentA::Cm*) resulted from a single-step transduction with a P1 lysate grown on *E. coli* OKN10::Cm. *E. coli* CAT4 (*ΔfepA, ΔcirA, Δfiu ΔfecA::Cm*) derived from 4 sequential transductions with lysates grown on *E. coli* strains OKN3::Cm, OKN5::Cm, OKN9::Cm and and OKN2::Cm, respectively. After each individual transduction we transformed the resulting strains with pCP20 and eliminated their chloramphenicol-resistance. The last transduction introduced *ΔfecA::Cm* into the chromosome, and we retained the resistance gene as a chromosomal marker for that strain. From *E. coli* CAT4 we generated *E. coli* CAT40 (*ΔfepA, ΔcirA, Δfiu, ΔfecA, entA::Cm*) by removing the chloramphenicol cassette from the *fecA* locus and transducing in *ΔentA::Cm* as described above. Finally, we confirmed the absence of *entA*, *tonB*, *fiu*, *fepA*, *fecA* or *cir* from the chromosome of *E. coli* strains CAT0, TonB, CAT4 and CAT40 by colony PCR reactions with primers ca. 500 bp upstream and downstream of the target gene. We also visualized the absence of the OM proteins from cell envelopes of *E. coli* CAT4 and CAT40 by SDS-PAGE, using cells grown in iron-deficient MOPS minimal media (Fig. 1).

**Table 1:** *E. coli* strains and plasmids.

Strain or Plasmid	Genotype
MG1655	wild type strain for colonization (CGSC 7740)
MG1655 Sm^r^	spontaneous Sm^r^ mutant of MG1655
MG1655 Sm^r^ Nal^r^	spontaneous Nal^r^ mutant of MG1655 Sm^r^
BN1071	*F-, rpsL trp, B1, entA,* [25]
BN1071/pKD46	starting strain for Datsenko mutagenesis [18]
OKN10::Cm	donor for *entA* [19]
OKN3::Cm	donor for *fepA* [19]
OKN2::Cm	donor for *fecA* [19]
OKN5::Cm	donor for *cir* [19]
OKN9::Cm	donor for *fiu* [19]
pKD3	template plasmid for cm-cassette amplification
pCP20	thermo-sensitive plasmid for excision of cm-cassette
CAT0	MG1655 Sm^r^ *entA::Cm*
CAT4	“ " *Δfiu ΔfepA ΔcirA ΔfecA::Cm*
CAT40	“ " " " " *ΔfecA entA::Cm*
*Δ*TonB	“ “ *ΔtonB::Cm*

### Bacterial growth

**Figure 2:**
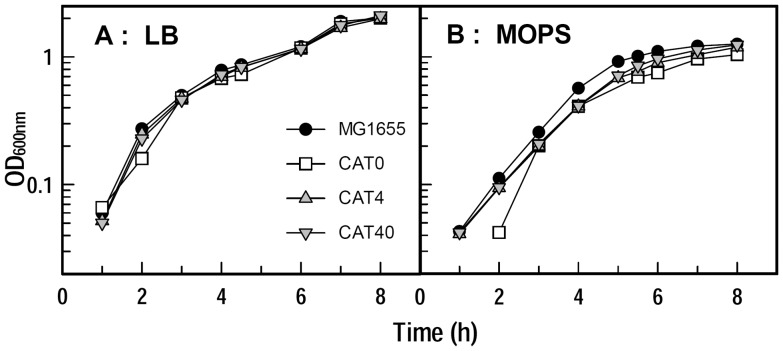
Bacterial growth curves in LB broth and iron-deficient MOPS medium. MG1655, CAT0 (*ΔentA*), CAT4 and CAT40 were grown in LB broth, subcultured at 1% into iron-deficient MOPS minimal media at 37°C, and growth in both media was spectroscopically monitored at 600 nm for 9–10 h.

Prior to their introduction into mice, we compared the growth of *E. coli* strains MG1655, CAT0, CAT4 and CAT40 in LB broth and iron-deficient MOPS minimal media for 9–10 hours (Fig. 2). None of the strains were impaired in LB broth, and grew with a doubling time of about 45 min. We also saw few differences in iron-deficient MOPS minimal medium, in which the typical doubling time was 75 min. *E. coli* CAT0 showed a lag in MOPS, but quickly reached the same growth rate and final cell density as the other strains. Thus the genetically engineered deletions did not generally impair bacterial growth.

### Colonization assays

The experiments compared chromosomally-encoded inabilities to synthesize (CAT0) or transport ferric catecholates (CAT4), as well as deficiencies in both enterobactin synthesis and ferric catecholate transport (CAT40), during colonization of the mouse intestine. Because conventional animals are completely resistant to colonization by enteric bacteria [21], the streptomycin-treated mouse model system was extensively used for studies of competitive intestinal colonization (see for example [21]–[23]). The mice are fed streptomycin in their drinking water for 24 h prior to colonization with streptomycin resistant *E. coli* strains [24]. The antibiotic pre-treatment selectively removes facultative anaerobes (e.g., *E. coli*, enterococci, streptococci, lactobacilli, and anaerobic lactobacilli and bifidobacteria) that are responsible for colonization resistance against *E. coli*, while leaving the anaerobic microbiota essentially intact. Hence the overall populations of anaerobes, including Bacteroides and Eubacterium, are unchanged following streptomycin treatment [25]. The streptomycin-treated mouse model allows colonization by experimental *E. coli* strains, and encompasses competition with large numbers of strict anaerobes. It is a system of choice for analysis of competition among *E. coli* strains in the intestine.

**Figure 3:**
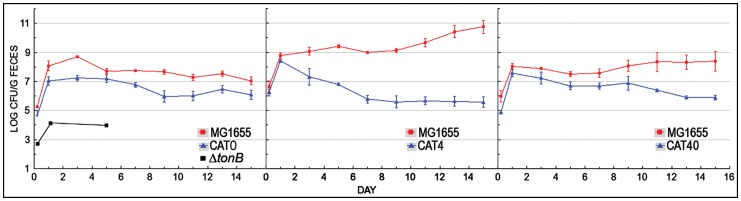
Colonization Competition between *E. coli* MG1655 and CAT0, CAT4 or CAT40. In each trial three streptomycin-treated mice were simultaneously fed with a mutant strain and wild type parent MG1655, which are both streptomycin resistant. If even a slight advantage exists between the two strains, the conditions of the large intestine select for the preferred strain, which dominates within a few days. If neither strain has an advantage, then the two strains co-colonize at almost equal levels. Fecal plate counts determined the relative colonizing abilities (the log difference in CFU/g of feces). Three-log differences or greater between the mutant and wild-type strain indicates in a major colonization defect. A 1.5 to 3 log difference shows a significant colonization defect, and a 1 to 1.5 log difference denotes a minor defect. Log differences less than 1 are not significant. In these experiments pairs of bacteria were orally inoculated into mice on day 0, and their presence in feces was monitored for 15 days. The plotted data represents the mean of two or more independent trials; error bars represent standard deviations of the means. *E. coli* MG1655 out-competed CAT0, CAT4 and CAT40 for colonization. Unexpectedly, CAT40 showed 1000-fold better persistence than CAT4, and maintained colonization at almost the same level as MG1655 for the first week.

In these studies we individually paired equal numbers of wild type *E. coli* (MG1655) and one of its mutant derivatives and fed the mixture to mice at a dose of 10^5^ CFU/animal. In each experiment we administered each strain to 3 mice and monitored bacterial persistence by plating limiting dilutions of fecal material on antibiotic-selective media over a 2-week period. At each time point we calculated the means and standard deviations of the means for each strain; we repeated each colonization experiment two or three times. Observations of the persistence of the strains *in vivo* (Fig. 3) showed that with the exception of the *ΔtonB::Cm* strain all the mutants initially colonized the mice at the same population size as MG1655, reaching almost the same level on day 1. However, CAT0, CAT4 and CAT40 declined afterward to 1-, 5- and 2-log lower levels, respectively, at day 15. CAT0 (*entA::Cm*) cannot synthesize enterobactin. CAT4 (*Δfiu, ΔfepA, ΔcirA, ΔfecA::Cm*) synthesizes enterobactin but cannot transport ferric catecholates. Its huge deficiency relative to MG1655 illustrated the fundamental need for iron transport during colonization of the gut. Furthermore, in competition with CAT4 the wild type strain ultimately reached a 10^2^-fold higher level than the 10^8^ cfu/g feces population size that is typical of *E. coli* MG1655 in streptomycin treated mice, suggesting that the presence of CAT4 facilitated its growth, probably by the latter strain's hypersecretion of enterobactin (see below). Next, it was noteworthy that CAT40 colonized mice better than CAT4. Strain CAT40 (*ΔfepA ΔcirA Δfiu ΔfecA, entA::Cm*) lacks both enterobactin synthesis and ferric catecholate uptake. Despite its additional inability to synthesize enterobactin, relative to CAT4, it colonized the mice better, to a level only 2-logs less than that of MG1655. Hence the loss of enterobactin biosynthesis actually increased the persistence of CAT40 ∼1000-fold relative to that of CAT4, in competitions with the wild type.

### Enterobactin secretion *in vivo*

**Figure 4:**
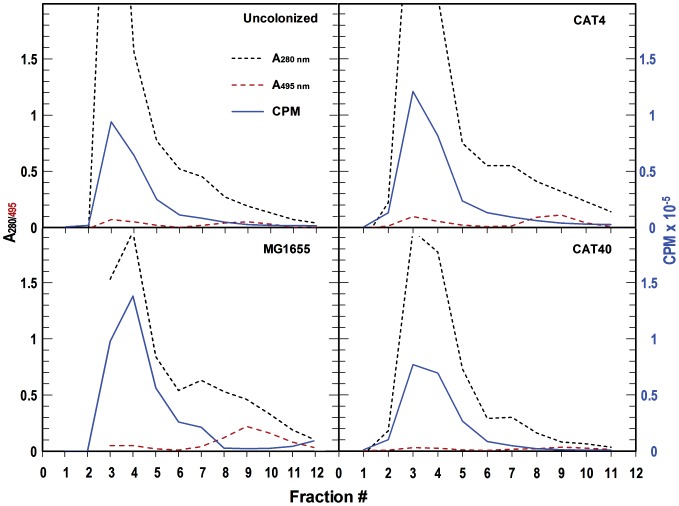
Enterobactin quantification in vivo. Mice were inoculated on day 0 and on day 12 caecal mucus from mice that were uncolonized or orally inoculated with *E. coli* strains MG1655, CAT4 or CAT40 was collected and diluted into 5 mM NaHPO_4_, pH 6.9. Samples from five individual mice in each experimental group were consolidated, the solution was clarified by centrifugation, 100 uL of each supernatant was mixed with 10 µCi of ^59^FeCl_3_, the samples were incubated on ice for an hour and then chromatographed on Sephadex LH20 (Materials and Methods). We chromatographed authentic FeEnt with the mucus as an internal marker, and determined and plotted the absorbances at 280 nm and 495 nm, and the radioactivity of each fraction. The black and red dashed lines show the absorbances of the eluted fractions at 280 nm and 495 nm, respectively: the blue line depicts their radioactivity.

**Figure 5:**
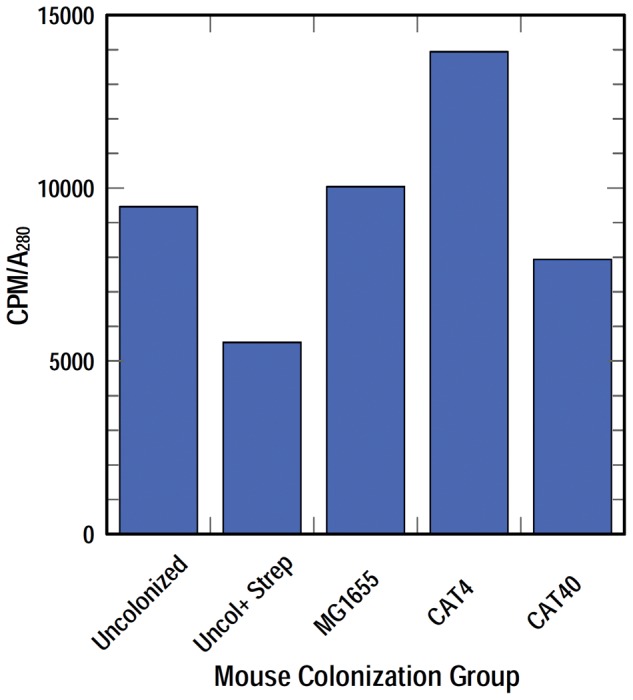
Summary of enterobactin production in vivo. The amount of FeEnt produced by the colonizing bacteria was standardized in relation to the amount of protein in the mucosal samples (CPM/A_280_ nm). The plotted data therefore depicts the relative amount of enterobactin production by each of the strains on day 12 after inoculation. This experiment was performed only once, but each data point represents the mean values from pooled extracts of 5 animals. Despite the fact that the cell numbers of *E. coli* CAT4 were 4-logs lower than those of MG1655 at this time, we found more ^59^FeEnt in the former strain's gut mucus. This finding, that CAT4 hyperexcretes enterobactin in vivo, corroborated previous findings *in vitro* [31], [32], [60], [61]. Even though they were present at 10,000-fold lower abundance (a finding that was substantiated by statistical analysis of three colonization experiments), CAT4 cells secreted as much or more enterobactin as wild type bacteria. This was validated by the background control, uncolonized mice treated with streptomycin, that established a baseline for the detection of FeEnt in the mice.

The better survival CAT40 than CAT4 in individual competition experiments with MG1655 suggested that the elimination of enterobactin synthesis relieved iron stress [26] *in vivo*. Enterobactin production was presumably detrimental in a genetic background lacking ferric catecholate transporters, because the high affinity aposiderophore sequestered iron from strains that could not transport it, rendering them fatally iron-deficient. To test this inference we performed a separate study in which we inoculated groups of mice with the single, individual *E. coli* test strains and measured the amount of enterobactin that they produced in the mouse gut. We performed this experiment only once (for an explanation see Materials & Methods), but the study analyzed pooled mouse gut lavage samples from groups that each contained five animals. For each reported value we consolidated material from 5 mice, so the determinations represent an intrinsic average. We evaluated five experimental groups: (1) conventional mice (no treatment; normal flora); (2) streptomycin-treated mice (un-inoculated with *E. coli*, but subjected to streptomycin in their drinking water to eliminate endogenous bacterial flora); (3) mice colonized by (wild type) MG1655; (4) mice colonized by CAT4; (5) mice colonized by CAT40. After inoculation we collected fecal samples and made bacterial counts on days 1, 5, and 11, and on day 12 we performed enterobactin concentration assays in cecal mucus samples from all the experimental groups (Materials and Methods). This procedure identified and quantified ferric catecholates in the mouse gut, because they uniquely chromatograph on Sephadex LH20 [27]. Once formed as [^59^Fe]-complexes, other siderophores (e.g, hydroxamates) and iron binding proteins (e.g., transferrin, lactoferrin, lipocalin) from the gut mucosa, which are more hydrophilic, pass faster through the hydrophobic resin and elute before ferric catecholates. In this experiment (Fig. 4) the red tracing depicts the reference elution peaks of exogenously added FeEnt: the first peak (fr. 3–5) derived from FeEnt that associated with host proteins (albumin [28], lipocalin [29], [30]), and the second peak (fr. 8–11) represented free FeEnt (as determined by chromatography of purified FeEnt on the same column; data not shown). The bulk of host protein (black dashed-line) eluted in fractions 3–7, and a smaller amount of hydrophobic UV-adsorbing material passed more slowly through the resin and eluted in fractions 8–11. We used the UV absorbance in fractions 6–7 as a measure of the total mucosal material to which [^59^Fe] was added, and related the eluted [^59^Fe]Ent (blue tracing; frs. 8–11) to this value (CPM_FeEnt_/A_280 mm_). This ratio (Fig. 5) gave a measure of the enterobactin produced in the guts of the different mouse groups.

In this light, the normal mouse microbiota produced a significant quantity of catecholate siderophores (untreated vs Sm-treated). MG1655 produced about the same amount of enterobactin as the wild flora, and CAT4 elaborated an even larger amount of enterobactin, despite the fact that its cell numbers were 4-logs lower than those of MG1655 on day 12. We found a 1.4-fold higher level of ^59^FeEnt in the CAT4 gut mucus, more than that produced by either the aggregate murine microbiota in the untreated mice or by MG1655-inoculated mice. Gram-negative bacteria that cannot transport enterobactin are well known to hyper-excrete it [31], [32], so the observation of this phenomenon *in vivo* was not surprising. Thus, the inability of the quadruple mutant CAT4 to transport ferric catecholates led to de-regulated enterobactin synthesis that starved the producing cells for iron, resulting in feeble intestinal colonization.

## Discussion

Microorganisms possess a variety of mechanisms to cope with restricted iron availability. Their approaches to acquire the metal in animals adapt according to the initial site of infection and their subsequent lifecycle in the host. Pathogens that multiply inside cells may access the iron-storage protein ferritin [33]. Mucosal surfaces are rich in lactoferrin, whereas transferrin predominates in blood and cerebrospinal fluid. Bacterial siderophores strip the iron from these eukaryotic proteins [28], [34] as a result of their generally much higher affinity for iron (III) (10^7^–10^26^ - fold higher). Or, bacterial cell surface receptor proteins may directly bind transferrin or lactoferrin and extract the metal [35]–[37]. Lastly, in blood and cerebrospinal fluid hemoglobin is the most abundant iron source in mammalian hosts, and bacterial pathogens contain systems to remove and capture its heme prosthetic group [12], [38]–[40]. Despite these abilities and mechanisms, conflicting reports exist about the relevance of siderophores in bacterial colonization and virulence. Previous experiments compared mouse infection by *Salmonella typhimurium* [41], or gut colonization by *E.coli* [42], [43] strains impaired in either enterobactin synthesis or FeEnt transport (*fepA, cir*), and concluded that these markers were inconsequential to host infection or colonization. However, in the former case the *S. typhimurium* strains retained all their ferric catecholate OM transporters, and in the latter the *E. coli* strain lacked only *fepA* and *cirA*: *fiu* and *fecA*, which are now known to transport ferric catecholates and their degradation products [44]–[46], remained. Other reports showed that siderophores are virulence determinants for *E. coli*, *Salmonella spp*, and *Klebsiella* [47]–[50].

Our findings resolve these conflicting data and eliminate any doubt about the importance of the native Gram-negative bacterial catecholate siderophores in bacterial colonization of animals. First, the *E. coli ΔtonB* did not colonize mice to any significant extent, suggesting the importance of Fe^3+^ uptake systems for establishment of a stable bacterial population in the gut. But, the well-known poor growth of *ΔtonB* strains in iron-restricted conditions complicates this result. The additional experiments described herein more directly evaluate iron acquisition in host colonization, by differentiating the influence of siderophore biosynthesis from ferric siderophore uptake. Inability to synthesize enterobactin alone only had a small impact on colonization, as evidenced by competitive colonization between MG1655 and CAT0 (*entA::Cm*). These data confirmed prior work with *S. typhimurium* [41]. In the absence of enterobaction production other forms of iron are available for absorption in the gut, including ferrous iron. But, these alternate systems are less efficient than iron supply by ferric catecholates, so a reduction in colonization occurred relative to the wild type strain. CAT4, on the other hand, that produces enterobactin but cannot transport ferric catecholates, showed a much more dramatic 5-log decrease in fecal counts, indicating a prodigious colonization defect. When nature's most potent iron-chelator, enterobactin, became an agent of iron-deprivation, bacterial colonization ceased. The 1000-fold reversal of this effect by elimination of enterobactin synthesis in CAT40 confirmed this interpretation.

We are aware of two previous reports attempting to measure the amount of enterobactin present in mammalian tissues. Der Vartanian et al, studying infection of lambs with *E. coli*, were unable to detect any enterobactin in the gut or any other biological material examined, even when the animals were inoculated with strains that produced enterobactin *in vitro* [51]. They did, however, find aerobactin in all the tissues they analyzed. A previous study measured enterobactin, salmochelin, and aerobactin production in chickens infected with a pathogenic *E. coli* strain, by homogenizing pericardia, air sacs, liver, and blood, and analyzing samples by liquid chromatography. Only chickens infected with high doses (10^8^) of bacteria by the intrathoracic air sac route had detectable levels of any siderophore. Their results indicate a shift towards higher production of aerobactin *in vivo*, with levels of enterobactin coming second, and salmochelin the lowest [52]. It is technically challenging to quantitatively measure the presence of small molecules like siderophores in the complex environment of the animal intestine. The radioisotopic approach that we employed was sensitive and discriminatory for the detection of FeEnt *in vivo*. The fact that untreated mice contained similar levels of catecholates to those found in mice colonized by MG1655 and the enterobactin overproducer CAT4 indicated that the normal microbiota actively synthesize the siderophores inside the gut environment [53]. Our experiments also show that strains unable to synthesize enterobactin (CAT0) can still colonize mice, likely because they transport the ferric catecholates formed when other bacteria synthesize enterobactin (e.g., MG1655). Conversely, *E. coli* strains devoid of ferric catecholate transporters were quickly outcompeted by the wild type *E. coli* MG1655, whether or not they can synthesize enterobactin. These data demonstrate that catecholates play a fundamental role in gut colonization by *E. coli*. Finally, the inability of CAT4 to transport ferric catecholates created a vicious cycle of iron deprivation and maximal de-repression of enterobactin synthesis, that further exacerbated the nutritional dilemma: overproduction of enterobactin effectively denied any iron acquisition by CAT4 *in vivo*, by complete chelation of free iron as FeEnt, which CAT4 cannot utilize.

Many pieces of evidence point to the relationship between iron and microbial pathogenesis. The presence of iron binding proteins like transferrin and lactoferrin in mammalian fluids and tissues restricts the concentration of free iron to low levels, as a part of the innate immune system. Both proteins bind the metal with strong affinity: the association constants for the equilibria between Fe^+++^ and transferrin or lactoferrin are 10^23^ M^−1^ [54] and 3×10^25^ M^−1^ [55], respectively. And, *in vivo* only 30 to 40% of the metal binding sites are saturated, so the proteins may sequester free iron that they encounter, reducing its availability to microorganisms. One immediate host response to bacterial infection is to further decrease the amount of available iron, so that the level of saturation of transferrin drops to less than 5% [16], [56]. Conversely, iron overload enhances susceptibility to bacterial infections, as illustrated by the finding that workers exposed to dust and fumes containing high concentration of iron showed increased rates of pneumonia and other respiratory infections [57]. Our results support these epidemiological correlations and establish the importance of ferric catecholate acquisition in Gram-negative bacterial host colonization.

## Materials and Methods

### Bacterial strains, plasmids and primers

All the bacteria used in this study were strains of *E. coli*. We previously constructed precise chromosomal deletions of known *E. coli* OM iron transporters (20) in strain BN1071 (Table 1). The deletion method replaced the target gene with a chloramphenicol-resistance cassette, with which we transferred the desired mutations from strains BN1071 to MG1655 by P1 transduction. Transformation of the resulting transductant strain with a thermo-sensitive plasmid that encoded a flippase gene (pCP20; [19]) removed the chloramphenicol cassette. To generate multiple mutations in a single strain we transduced deletions one-by-one into MG1655 Sm^r^ [58] and each time excised the drug resistance from the individual transductants. Then we repeated the process for the next deletion, until we accumulated all the desired mutations in MG1655 Sm^r^. After transduction of the last deletion we retained its chloramphenicol cassette, to mark the strain in competition experiments with prototrophic MG1655 Sm^R^. We propagated these strains in LB with appropriate antibiotics (streptomycin at 100 ug/ml, chloramphenicol at 20 ug/ml, ampicillin at 100 ug/ml). For isolation of bacteria from fecal samples we diluted the feces in Tryptone broth and plated the bacteria on MacConkey agar plates containing appropriate antibiotics.

### P1 transduction

An overnight culture of the donor strain (e.g., BN1071 *ΔentA::Cm^r^*) was diluted 1∶100 in fresh LB with 5 mM CaCl_2_ and 0.2% glucose, and shaken at 250 rpm for 1 h at 37°C. 100 uL of P1 phage lysate was added and the culture was shaken at 37°C until lysis (1 to 3 h). After adding a few drops of chloroform to the lysate, we sedimented debris by centrifugation, collected supernatant and stored it at 4°C. For transduction, the recipient strain (e.g., MG1655) was grown overnight in LB medium, harvested by centrifugation and resuspended in the same volume of LB containing 100 mM MgSO_4_ and 5 mM CaCl_2_. We mixed 100 uL aliquots of the cell suspension with 100 uL of undiluted or diluted (10-fold) P1 lysate, and incubated at 37°C for 1 hour without shaking. After interrupting the transductions with 100 mM sodium citrate, pH 5.5, we spread the cells on LB plates with chloramphenicol (20 ug/mL), and analyzed putative transductant colonies by PCR to confirm their chromosomal deletions.

### *In vitro* growth rates

Strains were grown overnight in LB broth and subcultured (1%) in fresh LB broth or iron-deficient MOPS minimal media [20]. After 2 h we began monitoring absorbance at 600 nm, and continued every 30 minutes thereafter for 12 h.

### Ethics statement

This study was performed in strict accordance with the recommendations in the Guide for the Care and Use of Laboratory Animals of the National Institutes of Health. The protocol was approved by the Institutional Animal Care and Use Committee of the University of Oklahoma, which has an Animal Welfare Assurance on file with the Office of Laboratory Animal Welfare (IACUC approval number: A-3240-01), effective through June 2013. To our knowledge, there is no *in vitro* experiment design that provides a reasonable alternative to the animal experiments. There is no discomfort to the animals during experiments and at the conclusion of experiments animals were sacrificed by CO_2_ asphyxiation. This method is consistent with recommendations of the Panel on Euthanasia of the American Veterinary Medical Association.

### Mouse colonizations

Three six week old CD-1 male mice were given streptomycin in their drinking water (5 g/L) for 24 h to eliminate the endogenous flora. The mice were starved for food and water for 24 h prior to ingestion of approximately 10^5^ colony forming units (CFU) of both *E. coli* MG1655 (Str^r^ Nal^r^) and a mutant strain (Sm^r^ Cm^r^) in 1 mL of 20% sucrose. The large intestine and the cecum are the main sites of bacterial colonization in streptomycin-treated mice [59], and the population sizes in the intestine are well represented in the feces [24]. After inoculation, food and streptomycin-water were restored *ad libitum* and fecal plate counts were determined at 5 h, 24 h, and on every other day thereafter for 15 days. Fecal samples were homogenized in 1% tryptone broth, serially diluted, and plated on MacConkey agar containing streptomycin (100 ug/mL) and nalidixic acid (50 ug/mL) to quantify the population of the wild type strain MG1655, as well as on MacConkey plus streptomycin and chloramphenicol (30 ug/mL) to determine the titer of the chromosomal deletion mutants. We repeated each colonization study at least twice; usually 3 times. The detection limit in fecal samples was 10^2^ CFU/g feces.

### Measurement of ferric enterobactin levels *in vivo*

Twelve days after inoculation with bacteria, mice were sacrificed by CO_2_ inhalation and gut mucus from the caecum of 3 mice in each group were combined, suspended in 200 uL of distilled water and chilled on ice. The suspensions were centrifuged at 10 K× g for 10 min at 4°C, and 10 uCi of ^59^FeCl_3_ was added to 100 uL of the clarified supernatant fluid. After incubation of the solution for 1 h on ice to allow formation of iron complexes, 0.25 uMol of ^56^FeEnt in 5 mM NaHPO_4_ pH 6.9 was added and the mixtures were individually chromatographed on 0.5×10 cm columns containing Sephadex LH20 in 5 mM NaHPO_4_, pH 6.9 [26]. 0.5 mL fractions were collected, subjected to spectroscopic analysis, and counted in a Packard Cobra gamma counter. Their absorbance at 280 nm gave a relative measure of protein concentration, and their red color and absorbance at 495 nm identified fractions containing ferric enterobactin. We plotted and the ratio of CPM in the (free) ferric enterobactin containing fractions 8–11 to the A_280 nm_ in fractions 6 and 7 (Fig. 4), in order to obtain a relative measure of enterobactin production in the guts of mice in the different experimental groups (Fig. 5).

We performed the in vivo catechol determination only once, but the study involved 5 groups that each contained 5 mice. The samples from each animal were too small to individually analyze for their catecholate content, even though we used the highest specific activity [^59^Fe] commercially available to make the determinations. Each reported data point derived from the pooled gut lavage samples of 5 mice, and therefore represents an intrinsic average of five individuals, which provides a high degree of reliability. In light of these considerations, and the fact that the observed catecholate levels *in vivo* recapitulated extensive previously published data [31], [32], [60], [61], we did not see the need to statistically re-validate a well-known phenomenon by the involvement of 25 additional animals.
